# Primary Barbed Suture Versus T‐Tube Drainage After Laparoscopic Common Bile Duct Exploration: A Randomized Controlled Trial

**DOI:** 10.1002/hsr2.72030

**Published:** 2026-03-10

**Authors:** Lipeng Niu, Kesai Yang, Yongtao Li

**Affiliations:** ^1^ Department of General Surgery, Affiliated Hospital of NCO School Army Medical University Shijiazhuang China

**Keywords:** common bile duct stones, gallbladder stones, laparoscopic common bile duct exploration, primary closure, T‐tube drainage

## Abstract

**Background and Aims:**

T‐tube drainage has traditionally been used after laparoscopic common bile duct exploration (LCBDE) to reduce the risk of bile leakage; however, it is associated with prolonged hospital stay and tube‐related complications. Primary closure of the common bile duct has emerged as an alternative strategy, but concerns regarding postoperative safety and patient selection criteria remain. This study aimed to compare perioperative outcomes between primary barbed suture closure and T‐tube drainage following LCBDE.

**Methods:**

This prospective single‐center randomized controlled trial enrolled patients with common bile duct stones undergoing LCBDE. Eligible patients were randomly assigned in a 1:1 ratio to either primary closure using a continuous barbed suture or conventional T‐tube drainage. Primary outcomes included operative time, postoperative drainage volume, length of hospital stay, and hospitalization costs. Secondary outcomes included postoperative complications. Statistical analyses were performed using appropriate independent‐sample tests and multivariable regression models, with effect sizes and 95% confidence intervals reported. Exploratory analyses assessed associations between common bile duct diameter and clinical outcomes.

**Results:**

A total of 45 patients were included in the final analysis (primary closure, *n* = 22; T‐tube drainage, *n* = 23). Baseline characteristics were comparable between groups. Compared with T‐tube drainage, primary closure was associated with shorter operative time, reduced postoperative drainage volume, shorter hospital stay, and lower hospitalization costs. No bile leakage or biliary stricture was observed in either group during the follow‐up period. Exploratory analyses suggested associations between common bile duct diameter and selected perioperative outcomes.

**Conclusion:**

Primary barbed suture closure after LCBDE was associated with favorable perioperative outcomes compared with T‐tube drainage, without an observed increase in early postoperative complications. Given the limited sample size, larger multicenter randomized controlled trials with extended follow‐up are needed to confirm long‐term safety.

**Abbreviations**
CBD: common bile duct
ERAS: enhanced recovery after surgery
LCBDE: laparoscopic common bile duct exploration
LOS: length of stay
MRCP: magnetic resonance cholangiopancreatography
OLS: ordinary least squares
PC: primary closure
TT: T‐tube drainage

## 1 Introduction

Choledocholithiasis is a common biliary disease that may lead to serious complications if not appropriately treated [1, 2, 3]. Laparoscopic common bile duct exploration (LCBDE) has become an effective minimally invasive approach for stone clearance and is increasingly performed as a single‐stage procedure [4, 5, 6]. Traditionally, T‐tube drainage has been used following LCBDE to decompress the biliary tree and reduce the risk of bile leakage [7]. However, T‐tube drainage is associated with tube‐related discomfort, prolonged hospital stay, electrolyte imbalance, and the risk of accidental tube dislodgement [8, 9, 10].

Primary closure of the common bile duct has been proposed as an alternative to T‐tube drainage, with the potential advantages of faster recovery and improved patient comfort [11, 12, 13]. Several randomized trials and meta‐analyses have suggested that primary closure may be associated with favorable perioperative outcomes without an increased risk of postoperative complications in selected patients [14, 15, 16]. A recent updated meta‐analysis further supports the use of primary closure compared with T‐tube drainage following LCBDE [17].

Despite these encouraging results, concerns remain regarding the safety of primary closure, particularly the risks of bile leakage and biliary stricture, as well as the optimal patient selection criteria [18, 19, 20, 21]. The present study aimed to compare perioperative outcomes between primary barbed suture closure and conventional T‐tube drainage following LCBDE in a prospective randomized controlled design.

## 2 Materials and Methods

### 2.1 Study Design and Patients

This prospective randomized controlled trial was conducted at a single tertiary center. Patients diagnosed with common bile duct stones and scheduled for LCBDE were screened for eligibility. Inclusion and exclusion criteria were applied as described previously [22]. The study was reported in accordance with the CONSORT guidelines.

Eligible patients were randomly assigned in a 1:1 ratio to either the primary closure group (PC group) or the T‐tube drainage group (TT group). Randomization was performed using a computer‐generated sequence. Block randomization was applied to maintain allocation balance throughout the enrollment period. As a result of the random block sequence and the absence of post‐randomization exclusions, the final distribution resulted in 22 patients in the PC group and 23 patients in the TT group. This slight numerical imbalance reflects random variation inherent to block allocation and does not indicate any deviation from the predefined randomization protocol. All randomized patients were included in the final analysis in accordance with the intention‐to‐treat principle. No patients were excluded after randomization.

The statistical methods used in this study were guided by the recommendations outlined in Assel et al. for reporting statistics in clinical research. We ensured that all results are reported with effect estimates, 95% confidence intervals, and *p* values where appropriate, following the SAMPL guidelines [23].

### 2.2 Surgical Technique

**Figure 1:**
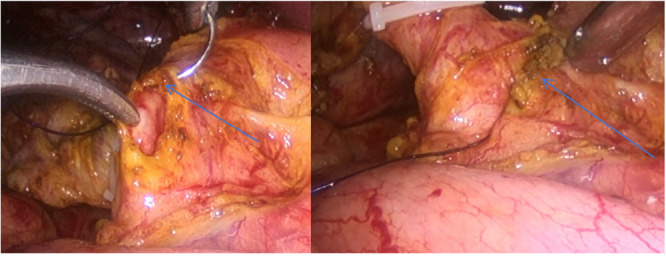
Schematic illustration of primary double‐layer barbed suture closure of the common bile duct after laparoscopic exploration.

All procedures were performed laparoscopically by the same experienced surgical team. After complete stone clearance was confirmed by choledochoscopy and saline irrigation, the common bile duct was closed primarily using a standardized double‐layer continuous barbed suture technique in the PC group (Figure 1). The inner mucosal layer was first approximated using a continuous barbed suture to restore luminal continuity and ensure watertight closure. Subsequently, an outer seromuscular continuous embedding layer was applied to reinforce the primary repair and provide additional structural support. Care was taken to avoid excessive tension during suturing in order to minimize the risk of luminal narrowing. In the TT group, a T‐tube was placed through the choledochotomy and allowed for external drainage. Details of the surgical procedure have been described previously [24, 25, 26].

### 2.3 Outcome Measures

Primary outcomes included operative time, postoperative drainage volume, length of hospital stay, and hospitalization costs. Secondary outcomes included postoperative complications such as bile leakage, biliary stricture, and other procedure‐related adverse events.

### 2.4 Statistical Analysis

Continuous data are expressed as mean ± standard deviation. Paired *t*‐tests were used exclusively for within‐patient pre‐ and postoperative comparisons, whereas all between‐group comparisons involved independent samples and were analyzed using Welch's *t*‐tests. Categorical variables were compared using chi‐square or Fisher's exact tests, as appropriate.

Pearson correlation analysis was used to explore linear associations between common bile duct diameter and clinical outcomes after assessment of linearity and approximate normality. These analyses were considered exploratory and hypothesis‐generating.

Separate multiple linear regression models were constructed for operative time, blood loss, length of hospital stay, and hospitalization costs, with each outcome modeled individually. Model assumptions, including linearity, homoscedasticity, and normality of residuals, were assessed prior to model fitting. Given the right‐skewed distribution of length of stay and hospitalization costs, log‐transformed models were additionally examined, yielding consistent directions and statistical significance of effect; results from the original scale are presented for clinical interpretability. The direction and statistical significance of associations remained materially unchanged in log‐transformed models (data not shown).

All statistical tests were two‐sided, and a *p* value < 0.05 was considered statistically significant. Statistical analyses were performed using SPSS version 26.0 (IBM Corp., Armonk, NY, USA).

### 2.5 Sample Size Calculation

An a priori sample size calculation was performed based on the primary endpoint of length of hospital stay. Based on prior institutional data and published literature, a mean difference of 3.0 days between groups was considered clinically meaningful, with an assumed standard deviation of 4.0 days. Using a two‐sided independent‐samples *t*‐test with a significance level (*α*) of 0.05 and a statistical power of 80% (1−*β* = 0.80), the minimum required sample size was estimated to be 21 patients per group. To account for potential variability and to preserve adequate statistical power, we aimed to enroll at least 22 patients in each group. The final analyzed cohort of 45 patients met this predefined requirement for detecting clinically meaningful differences in the primary endpoint.

## 3 Results

### 3.1 Baseline Characteristics

A total of 45 patients were included in the final analysis, with 22 patients in the PC group and 23 patients in the TT group. Baseline demographic and clinical characteristics were comparable between groups (Table 1).

**Table 1:** Baseline characteristics of patients.

Characteristic	PC (*n* = 22)	TT (*n* = 23)	Mean difference (95% CI)	*p* value
Age (years)	60.64 ± 17.40	66.43 ± 14.32	−5.79 (−15.63 to 4.05)	0.23
Preoperative total bilirubin (µmol/L)	42.93 ± 39.45	94.70 ± 115.61	−51.77 (−104.14 to 0.59)	0.05
CBD diameter (mm)	11.72 ± 4.11	13.13 ± 4.94	−1.41 (−4.17 to 1.35)	0.30
Female sex, *n* (%)	13 (59.1)	8 (34.8)	—	0.10
Hypertension, *n* (%)	5 (22.7)	5 (21.7)	—	1.00

### 3.2 Perioperative Outcomes

Compared with the TT group, the PC group demonstrated a trend toward shorter operative time and significantly reduced postoperative drainage volume, shorter length of hospital stay, and lower hospitalization costs (Table 2), with effect estimates and 95% confidence intervals presented to facilitate clinical interpretation.

**Table 2:** Perioperative outcomes.

Outcome	PC (*n* = 22)	TT (*n* = 23)	Mean difference (95% CI)	*p* value
Operative time (min)	103.41 ± 30.25	129.13 ± 54.74	−25.72 (−52.91 to 1.47)	0.06
Blood loss (mL)	33.86 ± 16.90	79.13 ± 85.12	−45.27 (−83.01 to −7.53)	0.02
Length of stay (days)	11.95 ± 4.40	15.26 ± 3.80	−3.31 (−5.77 to −0.85)	0.01
Hospital cost (CNY)	19,832 ± 2647	26,056 ± 7631	−6224 (−10,111 to −2337)	0.001
Drainage volume (mL)	48.86 ± 63.09	114.78 ± 106.19	−65.92 (−118.50 to −13.35)	0.02

### 3.3 Postoperative Bilirubin Recovery

Postoperative bilirubin levels were comparable between the two groups, although the absolute reduction in total bilirubin was greater in the T‐tube group, likely reflecting higher baseline values (Table 3).

**Table 3:** Postoperative bilirubin recovery.

Parameter	PC	TT	Mean difference (95% CI)	*p* value
Total bilirubin postop (µmol/L)	18.9 ± 10.2	22.6 ± 15.8	−3.7 (− 11.8 to 4.4)	0.35
Δ Total bilirubin	−24.0 ± 33.2	−72.1 ± 115.4	48.1 (3.0 to 93.2)	0.04
Δ Indirect bilirubin	−8.8 ± 10.3	−21.7 ± 24.6	12.9 (1.0 to 24.8)	0.03

### 3.4 Postoperative Complications

No bile leakage, biliary stricture, or major postoperative complications were observed in either group during the follow‐up period.

### 3.5 Exploratory Analyses

Exploratory analyses suggested associations between common bile duct diameter and selected perioperative outcomes (Table 4). After adjusting for potential confounders, primary closure remained independently associated with shorter hospital stay and lower costs in multiple linear regression models (Table 5). Subgroup analyses based on a CBD diameter threshold of 8 mm are presented in Table 6; however, these findings should be interpreted with caution given the limited sample size.

**Table 4:** Exploratory Pearson correlation with CBD diameter.

Outcome	*r*	95% CI	*p* value
Operative time	0.13	−0.17 to 0.41	0.39
Blood loss	0.10	−0.20 to 0.38	0.53
Length of stay	0.21	−0.09 to 0.48	0.16
Hospital cost	0.29	−0.01 to 0.52	0.06

**Table 5:** Multiple linear regression analysis.

Outcome	*β* (PC vs. TT)	95% CI	*p* value
Operative time	−29.03	−67.32 to 9.26	0.13
Blood loss	−4.03	−24.55 to 16.49	0.69
Length of stay	−2.67	−5.33 to −0.01	0.05
Hospital cost	−5073.9	−8612.4 to −1535.4	.007

**Table 6:** Subgroup analysis by CBD diameter (exploratory).

Outcome	CBD ≥ 8 mm: mean difference (95% CI)	*p* value
Blood loss (mL)	−45.02 (−86.39 to −3.65)	0.03
Length of stay (days)	−2.99 (−5.63 to −0.35)	0.03

## 4 Discussion

In this prospective randomized controlled trial performed by a single experienced surgeon under standardized operative conditions, primary double‐layer barbed suture closure of the common bile duct (CBD) after laparoscopic common bile duct exploration (LCBDE) was associated with less intraoperative blood loss, shorter postoperative hospital stay, lower postoperative drainage volume, and reduced hospitalization costs compared with T‐tube drainage, without an observed increase in early postoperative complications. Operative time also demonstrated a trend toward reduction. Postoperative bilirubin recovery was comparable between the two groups, and no bile leakage or bile duct strictures were observed during follow‐up; the greater absolute reduction in bilirubin observed in the T‐tube group likely reflects higher baseline bilirubin levels rather than delayed recovery in the primary closure group. These findings are consistent with previous randomized controlled trials and long‐term follow‐up studies supporting the safety and clinical advantages of primary closure when performed under appropriate technical conditions [16, 22].

After multiple linear regression adjustment, primary closure remained associated with shorter hospitalization and lower costs, suggesting that surgical technique and operator experience may play a more decisive role than a strict diameter cutoff [26, 27].

Endoscopic retrograde cholangiopancreatography with endoscopic sphincterotomy (ERCP/EST) remains an important therapeutic option in the management of choledocholithiasis; however, its endoscopy‐related complications—including pancreatitis, cholangitis, hemorrhage, and perforation—are well documented [10, 11, 12]. In addition, ERCP/EST may impair sphincter of Oddi function and is associated with long‐term consequences such as duodenobiliary reflux and stone recurrence [11, 12]. For patients suitable for LCBDE, single‐stage stone clearance combined with primary closure avoids secondary procedures, reduces total hospitalization time and costs, and better aligns with enhanced recovery after surgery (ERAS) principles [7, 13]. Although alternative drainage strategies such as antegrade or internal stents have been described, they do not fundamentally eliminate the limitations associated with staged management or device‐related complications [14, 15]. In centers with sufficient technical expertise, LCBDE combined with primary closure therefore represents a rational and evidence‐supported strategy [7, 10, 13, 15, 22].

Compared with T‐tube drainage, primary closure offers several mechanistic and practical advantages. First, it eliminates tube‐related procedural steps and device‐specific complications, including tube dislodgement, inadequate drainage, cholangitis after removal, and bile peritonitis [7, 28]. Second, primary closure shortens hospitalization and reduces overall treatment costs by avoiding postoperative T‐tube cholangiography, prolonged external drainage, and delayed tube removal. Prior studies and economic analyses have consistently demonstrated advantages in length of stay and cost‐effectiveness associated with primary closure [7, 13]. Third, primary closure more closely preserves physiological bile flow, preventing prolonged external bile loss and potential electrolyte imbalance. The significantly lower postoperative drainage volume observed in the primary closure group in the present study is consistent with this physiological mechanism.

The use of double‐layer continuous barbed suturing further refines the technical execution of primary closure. Barbed sutures provide knot‐free placement, self‐locking tension, uniform force distribution, and improved efficiency, making them particularly suitable for closure of narrow luminal structures such as the CBD [18, 20, 21, 29]. The absence of knot tying may reduce focal ischemia or tissue strangulation in confined operative fields. In this study, a dual‐layer suturing strategy was adopted. The inner mucosal continuous suture restores luminal continuity and re‐establishes a smooth mucosal barrier, thereby potentially reducing bile leakage risk [30]. The outer seromuscular continuous embedding layer provides additional reinforcement and compensates for potential instability associated with bile duct edema or tissue fragility. The combination of dual‐layer continuity and the uniform self‐locking tension of barbed sutures likely contributes to reduced microleakage and explains the absence of bile leakage and the lower postoperative drainage volume observed in this cohort. Previous studies have similarly demonstrated the safety and feasibility of barbed suture closure during LCBDE [31, 32, 33]. Under a standardized operative pathway, the present findings further support the reproducibility and clinical utility of this approach.

Operator variability represents an important source of heterogeneity in surgical research and may obscure true treatment effects [7]. Systematic reviews of LCBDE have noted that differences in suturing technique and operative strategy contribute substantially to inter‐study variability [7]. By employing a single experienced surgeon for all procedures, this study minimized operator‐related confounding and allowed a clearer assessment of the technical pathway itself [16]. Although multicenter randomized trials provide broader generalizability, they may dilute technique‐specific effects due to inter‐surgeon variability in suture selection, layer construction, and tension control [16, 22]. The observed reductions in blood loss, length of stay, drainage volume, and hospitalization costs in the present study can therefore be more confidently attributed to the standardized double‐layer barbed suture technique rather than to differences in surgeon proficiency [7, 16, 22]. Nevertheless, validation in multicenter settings remains necessary to confirm external applicability.

The clinical relevance of common bile duct diameter remains an area of ongoing discussion. A diameter threshold of ≥ 8 mm has been proposed as a practical criterion for safe primary closure in several systematic reviews [26]. However, single‐center series have demonstrated that primary closure of normal‐caliber bile ducts can be performed safely when complete stone clearance and meticulous suturing are achieved [27, 30]. In the present study, CBD diameter was not significantly correlated with operative time, blood loss, or length of hospital stay. After multiple linear regression adjustment, primary closure remained associated with shorter hospitalization and lower costs, suggesting that surgical technique and operator experience may play a more decisive role than a strict diameter cutoff [26, 27]. Given the limited sample size, particularly within subgroup analyses, findings related to CBD diameter should be interpreted cautiously and regarded as exploratory and hypothesis‐generating rather than definitive.

Several strengths and limitations merit consideration. The use of single‐surgeon consecutive procedures reduced operator variability and enhanced internal validity. The standardized double‐layer barbed suturing protocol provides a clearly defined and reproducible technical framework. However, although the sample size was sufficient to detect clinically meaningful differences in primary endpoints, the study was not powered to detect rare adverse events such as bile leakage or late biliary stricture. This study should also be interpreted as a preliminary randomized controlled trial designed to evaluate the feasibility and potential clinical benefits of standardized double‐layer barbed suture closure following LCBDE. Although the sample size was limited, the findings provide initial effect estimates and feasibility data that may inform the design and sample size calculation of future adequately powered multicenter randomized trials. Confirmation in larger cohorts is necessary before definitive conclusions regarding rare adverse events and long‐term safety can be established. Furthermore, the single‐center design may limit generalizability despite improving internal consistency. Finally, the 6‐month follow‐up period is adequate for early postoperative outcomes but insufficient for assessing long‐term sequelae such as late biliary stricture or stone recurrence.

In summary, under experienced operators and standardized technical protocols, primary double‐layer barbed suturing of the CBD after LCBDE was associated with favorable perioperative outcomes compared with T‐tube drainage in terms of blood loss, length of hospital stay, postoperative drainage volume, and hospitalization costs, without an observed increase in early complications. When interpreted within the limitations of sample size and follow‐up duration, these findings support the role of primary closure as an effective strategy in appropriately selected patients, while underscoring the importance of surgical expertise and technical standardization in optimizing outcomes.

## 5 Conclusion

Under experienced operators and standardized technical protocols, primary double‐layer barbed suturing of the CBD after LCBDE was associated with favorable perioperative outcomes compared with T‐tube drainage, including reduced blood loss, shorter length of hospital stay, lower postoperative drainage volume, and decreased hospitalization costs, without an observed increase in early postoperative complications in this cohort.

Given the limited sample size and follow‐up duration, particularly with respect to rare adverse events, these findings should be interpreted with appropriate caution. Larger multicenter randomized controlled trials with longer follow‐up are warranted to confirm long‐term safety and further refine patient selection criteria.

Within the context of these limitations, primary closure using a standardized double‐layer barbed suture technique may represent a promising alternative to T‐tube drainage in appropriately selected patients.

## Author Contributions

**Lipeng Niu:** writing – original draft preparation, data curation, formal analysis. **Kesai Yang:** data curation, formal analysis, methodology. **Yongtao Li:** conceptualization, investigation (surgical performance), supervision, writing – review and editing.

## Ethics Statement

This study was approved by the Ethics Committee of the Affiliated Hospital of NCO School, Army Medical University (Approval No. LFSYY2025008). All procedures performed in studies involving human participants were conducted in accordance with the ethical standards of the institutional and/or national research committee and with the 1964 Helsinki Declaration and its later amendments or comparable ethical standards. Written informed consent was obtained from all individual participants included in the study.

## Conflicts of Interest

The authors declare no conflicts of interest.

## Transparency Statement

The corresponding author, Yongtao Li, affirms that this manuscript is an honest, accurate, and transparent account of the study being reported; that no important aspects of the study have been omitted; and that any discrepancies from the study as planned (and, if relevant, registered) have been explained.

## Supporting information

Supplementary_Video_1

## Data Availability

The data that support the findings of this study are available on request from the corresponding author. The data are not publicly available due to privacy or ethical restrictions.
